# The combined association of adherence to recommended physical activity and glycemic control with depression: an exploratory study with mediation and moderation models

**DOI:** 10.1186/s12889-023-17063-y

**Published:** 2023-11-02

**Authors:** Yuchuan Zou, Fanchao Meng, Xiuping Yan

**Affiliations:** 1 The National Clinical Research Center for Mental Disorders & Beijing Key Laboratory of Mental Disorders, Beijing Anding Hospital, Capital Medical University, 100088 Beijing, China; 2 Advanced Innovation Center for Human Brain Protection, Capital Medical University, 100088 Beijing, China

**Keywords:** Physical activity, Glycemic control, Depression, NHANES

## Abstract

**Background:**

Both physical activity and dysglycemia are associated with depression. However, the combined association of adherence to recommended physical activity (RPA) and glycemic control with depression is unknown. Moreover, the extent to which glycemic control mediates the association between physical activity and depression is not established.

**Methods:**

The sample included 31,302 adults from the National Health and Nutrition Examination Survey 2007-08 to 2017-18. Adherence to RPA for aerobic activity was defined according to the WHO 2020 guidelines. HbA1c was classified as < 5.7%, 5.7–6.4%, 6.5–6.9%, and ≥ 7.0%. Depression was evaluated according to the 9-item Patient Health Questionnaire. The odds ratio for depression stratified by adherence to RPA and HbA1c level were estimated by logistic regressions. Mediation analysis was performed to estimate the direct associations (not through glycemic control) and indirect associations (through glycemic control).

**Results:**

A total of 2871 participants were diagnosed with depression. Compared to participants with HbA1c level < 5.7% who adhere to RPA, those with HbA1c level < 5.7%, 5.7-6.4%, 6.5-6.9%, and ≥ 7.0% who did not adhere to RPA had increased odds ratio for depression, especially in women and older adults. Individuals with HbA1c ≥ 7.0% still had an increased odds ratio for depression even though they were physically active. The results of the mediation analysis were insignificant.

**Conclusion:**

There was a combined association of adherence to RPA and glycemic control with depression in women and older adults. We did not find out evidence of glycemic control mediation on the pathway from physical activity to depression.

## Introduction

Depression is a common mental disease worldwide and the number of people suffering from depression was increasing rapidly during the recent decades [1]. It was estimated that more than 280 million people around the world were suffering from depression, causing more than 47 million disability-adjusted life years in 2019 [2]. Patients with depression barely receive adequate treatment and there is a high risk of relapse after first-line treatment. Effective interventions are required for the prevention and management of depression.

Physical activity, referring to any movement by the skeletal muscles that require energy expenditure above that of rest, is important to improve health. Several guidelines have been issued in order to reduce levels of physical inactivity in adults and adolescents [3–7]. The latest guidelines emphasized that adults should do at least 150 to 300 min a week of moderate-intensity, or 75 to 150 min a week of vigorous-intensity aerobic physical activity, or an equivalent combination of both [3, 4].

Physical activity has beneficial effects on depression and could be used to reduce depressive symptoms in a therapeutic way [8, 9]. It is consistently reported as an effective intervention for the prevention of depression [10]. For example, one meta-analysis of 49 prospective studies found that people with low levels of physical activity had 17% [95% confidence interval (CI), 12-21%] higher odds ratio (OR) of developing depression compared to those with high levels of physical activity [11]. In a recent meta-analysis, investigators found that adults who achieved the recommended volume of physical activity had a 25% (95% CI, 18-32%) lower risk of depression compared to those who did not report any activity [12]. Thus, physical activity was recommended for the prevention of depression [3, 12].

Dysglycemia, referring to an abnormality in blood sugar stability including hypoglycemia or hyperglycemia, is an established risk factor for depression and glycemic control is important for the management of depression [13, 14]. Several biological mechanisms related to brain function, neurochemistry, and brain structure have been proposed for why dysglycemia would lead to the development of depression [15]. For example, a review study suggested that the effect of diabetes on depression was regulated by impaired hippocampal neurogenesis, dendritic remodeling, and increased apoptosis [16]. The pathological changes in the brain caused by hyperglycemia lead to the failure of maintaining learning and memory and governing emotional expression [16]. An animal study found that diabetes-related depression was associated with structural and functional damage to the neurovascular unit [17]. The role of hypothalamic-pituitary-adrenal axis dysfunction had also been indicated in the relationship between hypoglycemia and depression [18].

Physical activity has proved to be the established factor for glycemic control. For example, according to a meta-analysis of randomized control studies, there were significant differences between the exercise and control groups regarding hemoglobin A1C improvement after exercise intervention [19]. The effects of physical activity on glycemic control were suggested to be mediated by multiple mechanisms such as glucose uptake, fat oxidation, or pancreatic function improvement [20].

However, the combined association of physical activity and glycemia control with depression is unclear. In addition, it is not clear whether the association between physical activity and depression was mediated through glycemic control. In this study, we aimed to investigate the joint association of adherence to recommended physical activity (RPA) and glycemic control with depression. The degree to which glycemia control mediates the association between adherence to RPA and depression was also quantified by mediation analyses.

## Methods

### Study population

This study used cross-sectional data from US National Health and Nutrition Examination Survey (NHANES). It is a nationally representative survey of noninstitutionalized, civilian, US citizens conducted continuously in 2-year cycles using a stratified, multistage probability design [21]. All participants completed a detailed in-home interview, followed by an examination consisting of medical and physiological measurements as well as laboratory tests at a mobile examination. The study protocol was approved by the National Center for Health Statistics, and all participants gave their informed written consent before participation in the examination. We used data from 2007 to 08 through 2017-18 as NHANES used a different questionnaire for the evaluation of physical activity before 2007.

### Measures and definitions

During the in-home interview, information on age, gender, race/ethnicity, education, income, history of cardiovascular disease (CVD), smoke, alcohol use, and drug use was collected by standardized questionnaires. Race/ethnicity was categorized as Mexican American, non-Hispanic White, non-Hispanic Black, and others. Educational level was classified as less than high school, high school graduate, and college or higher. Income-to-poverty ratio, which was defined as annual family income divided by the poverty threshold adjusted for family size and inflation, was categorized as less than 1.3 and 1.3 or above. Participants were categorized as current smokers or not according to questions about whether they were currently smoking. History of CVD (including stroke, congestive heart failure, angina, and myocardial infarction), was determined by self-report. Current excessive alcohol use was defined as drinking 5 drinks/day or more on average in the 12 months prior to the interview. Antidepressant use was evaluated using the second level of drug ingredient categorical codes. A combination of bupropion and naltrexone was excluded as it was primarily used for weight-related medical problems.

During the physical examination, weight and height were measured to calculate the body mass index (BMI: weight in kilograms divided by height in meters squared). Blood pressure (BP) was also measured by trained staff and the mean BP was calculated as the mean of three or four readings. Blood samples were collected during the examination and sent to the central laboratories for the measurement of fasting insulin, fasting glucose, HbA1c, and total cholesterol. Important medical disease comorbidities that might affect depression including hypertension, diabetes, and dyslipidemia were identified. Hypertension was determined as systolic BP of 130 mm Hg or higher, diastolic BP of 80 mm Hg or higher, or the use of antihypertensive drugs; diabetes as HbA1c of 6.5% or higher, or the use of antidiabetic drugs; dyslipidemia as total cholesterol of 240 mg/dl or higher, or the use of lipid-lowering drugs. Insulin resistance (HOMA-IR) was determined by the formula developed by Mathews et al. as (fasting insulin [µU/mL]) × (fasting glucose [mmol/L]/22.5) [22].

Depressive symptoms were evaluated by the 9-item Patient Health Questionnaire (PHQ-9) according to the Diagnostic and Statistical Manual of Mental Disorders (Fourth edition). The frequency of depressive symptoms over the last two weeks was collected. Participants with higher scores tend to have more severe depression. However, for the convenience of description, we used.

a summed score on the PHQ-9 of 10 points or greater to define depression. A PHQ-9 score ≥ 10 had a favorable balance between sensitivity and specificity [23]. Physical activity was evaluated by the Global Physical Activity Questionnaire [24]. It is a validated tool that assesses leisure-time, occupation-related, and transportation-related physical activity [25, 26]. For leisure-time and occupation-related physical activity, questions were asked about the intensity, frequency, and duration of a typical week. For the transportation-related physical activity, questions were asked about the number of days in a typical week and the mean duration per day that they participated in the activity. The total duration of physical activity was determined as the minutes of moderate-intensity activity plus twice the minutes of vigorous-intensity activity of all three domains. Participants who had 150 min of physical activity or more per week were classified as adhering to RPA.

### Statistical analyses

In this study, participants with missing information on depression were excluded. Moreover, in the univariate analysis, participants with missing information were excluded for each variable. The baseline characteristics of participants were stratified by participants with and without depression. Continuous variables were expressed as mean (standard error) and categorical variables as proportion (standard error). In the univariate analysis, differences between participants with and without depression were assessed using independent Student’s t tests and Chi-square tests. HbA1c level was classified as < 5.7%, 5.7–6.4%, 6.5–6.9%, and ≥ 7% [27]. OR and 95% CI for depression stratified by adherence to RPA and HbA1c level were estimated by multivariable logistic regressions. Individuals who adhere to RPA and had HbA1c level < 5.7% were used as the reference. The models were adjusted for age, gender, race/ethnicity, education, income, BMI, hypertension, dyslipidemia, history of CVD, smoke, alcohol use, antidepressant use, and HOMA-IR.

In the mediation analysis, we aimed to quantify the degree to which glycemic control mediates the association between adherence to RPA and depression. We assessed the total, direct, and indirect effects of adherence to RPA on depression with glycemic control as a mediator. The mediated proportion was calculated as the estimated indirect effect size divided by the estimated total effect size. The aforementioned covariates were adjusted in the regression model. A more detailed description has been published previously [28].

In the NHANES, a complex, multistage, probability sampling design is used to select participants representative of the civilian, non-institutionalized US population. It employs a complex 4-stage design to have a representative sample and oversampling of certain population subgroups is intentionally done to increase the precision for subgroup estimates [21]. To ensure unbiased calculation, statistical analysis has accounted for the complex survey design of NHANES by incorporating primary sampling units, special sample weights, and strata according to the recommendations of the National Center for Health Statistics [29]. The primary sampling units and strata were provided directly in the NHANES, and appropriate 12-year sample weights were constructed by dividing the raw weights with the total number of survey cycles. Subgroup analyses were conducted according to gender (male and female) and age (< 45 and ≥ 45) as the odds of depression differed significantly by gender and age [30, 31]. Statistical analyses were conducted in R version 4.1.3 (R Foundation for Statistical Computing, Vienna, Austria). All statistical tests were 2-sided and considered significant at P < 0.05.

## Results

A total of 57,414 participants had the detailed in-person interview and physical examination from NHANES 2007-08 to 2017-18 and 35,162 of them were adults aged 18 years or older. Among 35,162 of them, 372 were excluded due to pregnancy and 3488 were excluded due to missing information on depression. Finally, a total of 31,302 individuals were included in the current study and 2871 of them were diagnosed with depression.

The baseline characteristics of participants with and without depression were presented in Table 1. Depressed individuals were less likely to be men and non-Hispanic White. They tend to receive less education and had lower income and higher BMI. They were more likely to have comorbidities (hypertension, diabetes, dyslipidemia, and CVD) and to engage in smoke, alcohol use, and antidepressant use. In addition, the depressed individuals were less likely to adhere to RPA and had a higher level of HbA1c and HOMA-IR.

**Table 1:** Baseline characteristics of participants with and without depression

Characteristics	Without depression (N = 28,431)	With depression (N = 2871)	P-value
Mean age, year	46.9 (0.3)	46.4 (0.4)	0.280
Men, %	50.6 (0.3)	36.3 (1.2)	< 0.001
Non-Hispanic White, %	67.1 (1.4)	63.0 (1.9)	0.003
Less than high school education, %	15.1 (0.6)	25.8 (1.2)	< 0.001
Income-to-poverty ratio < 1.3, %	13.5 (0.5)	29.7 (1.3)	< 0.001
BMI	28.9 (0.1)	30.6 (0.2)	< 0.001
Hypertension, %	45.6 (0.6)	50.3 (1.3)	0.001
Diabetes, %	11.1 (0.3)	17.8 (1.0)	< 0.001
Dyslipidemia, %	28.3 (0.4)	33.1 (1.1)	< 0.001
History of CVD	8.1 (0.2)	16.9 (0.9)	< 0.001
Current smoke, %	17.8 (0.4)	39.0 (1.4)	< 0.001
Excessive alcohol use, %	10.6 (0.4)	13.3 (0.8)	< 0.001
Antidepressant use, %	19.7 (0.5)	50.3 (1.6)	< 0.001
HOMA-IR	4.2 (0.1)	5.4 (0.2)	< 0.001
Adherence to RPA, %	66.5 (0.5)	52.4 (1.9)	< 0.001
Level of HbA1c, %	5.6 (1.0)	5.8 (2.6)	< 0.001

All the data were presented as mean or percentage with standard error in parenthesis

CVD: cardiovascular disease; RPA: recommended physical activity; HOMA-IR: insulin resistance

**Fig. 1:**
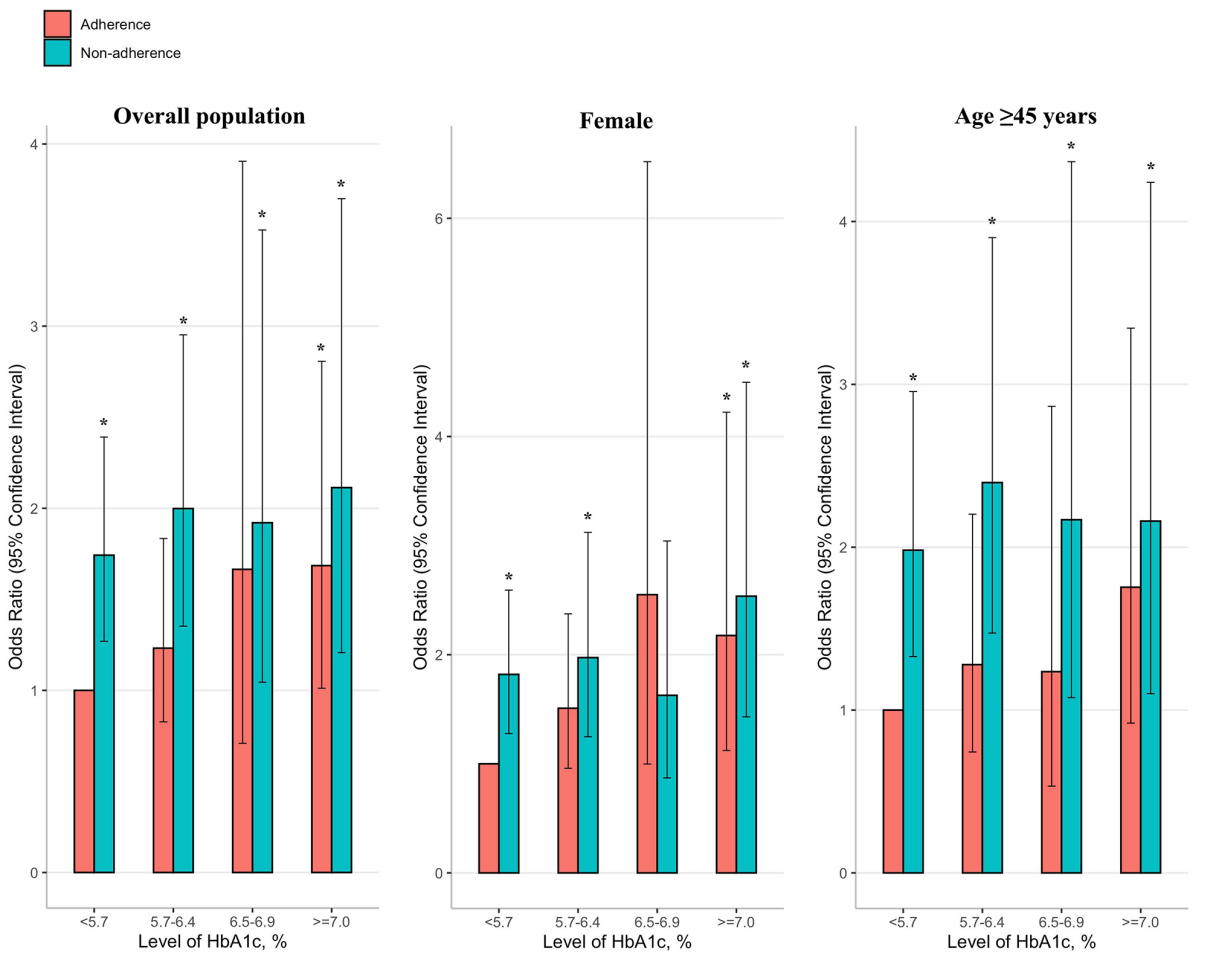
The combined association of adherence to recommended physical activity and glycemic control with depression among different populations. Models were adjusted for age, gender, race/ethnicity, education, income, body mass index, hypertension, dyslipidemia, history of cardiovascular disease, smoke, alcohol use, antidepressant use, and insulin resistance. Individuals who adhere to recommended physical activity and had a HbA1c level < 5.7% were used as the reference. *Significantly different from the reference

Table 2; Fig. 1 present the joint association of physical activity and glycemic control with depression. Compared to participants with HbA1c level < 5.7% who adhere to RPA, those with HbA1c level < 5.7%, 5.7-6.4%, 6.5-6.9%, and ≥ 7.0% who did not adhere to RPA had an increased OR for depression [The OR (95% CI) was 1.7 (1.3–24), 2.0 (1.4-3.0), 1.9 (1.0-3.5), and 2.1 (1.2–3.7), respectively]. In addition, for individuals with HbA1c level ≥ 7.0%, they still had an increased OR for depression even though they were physically active (OR, 1.7; 95% CI, 1.0-2.8). We performed subgroup analyses according to gender and age. The results showed that the combined association of physical activity and glycemic control with depression among females and individuals with age ≥ 45 years was generally similar to that among the overall population (Table 2; Fig. 1).

**Table 2:** OR (95% CI) of depression for participants with different level of HbA1c who adhere or did not adhere to the RPA

RPA	Level of HbA1c	Overall population	Male	Female	Age < 45 years	Age > = 45 years
Adherence	< 5.7%	Reference	Reference	Reference	Reference	Reference
Non-adherence	< 5.7%	**1.7 (1.3–2.4)**	1.7 (0.9–3.3)	**1.8 (1.3–2.6)**	1.5 (0.9–2.4)	**2.0 (1.3-3.0)**
Adherence	5.7-6.4%	1.2 (0.8–1.8)	0.9 (0.5–1.8)	1.5 (1.0-2.4)	1.3 (0.6–2.4)	1.3 (0.7–2.2)
Non-adherence	5.7-6.4%	**2.0 (1.4-3.0)**	**2.2 (1.2–3.9)**	**2.0 (1.2–3.1)**	1.1 (0.4–2.6)	**2.4 (1.5–3.9)**
Adherence	6.5-6.9%	1.7 (0.7–3.9)	**0.1 (0.0-0.5)**	2.6 (1.0-6.5)	4.3 (0.7–2.7)	1.2 (0.5–2.9)
Non-adherence	6.5-6.9%	**1.9 (1.0-3.5)**	2.3 (0.8–6.4)	1.6 (0.9-3.0)	**0.0 (0.0–0.0)**	**2.2 (1.1–4.4)**
Adherence	≥ 7.0%	**1.7 (1.0-2.8)**	1.3 (0.6-3.0)	**2.2 (1.1–4.2)**	1.4 (0.6–3.3)	1.8 (0.9–3.3)
Non-adherence	≥ 7.0%	**2.1 (1.2–3.7)**	1.7 (0.6–4.7)	**2.5 (1.4–4.5)**	2.7 (0.6–1.1)	**2.2 (1.1–4.2)**

RPA: recommended physical activity; OR: odds ratio; CI: confidence interval

Participants with HbA1c level < 5.7% who adhere to RPA were taken as the reference

**Fig. 2:**
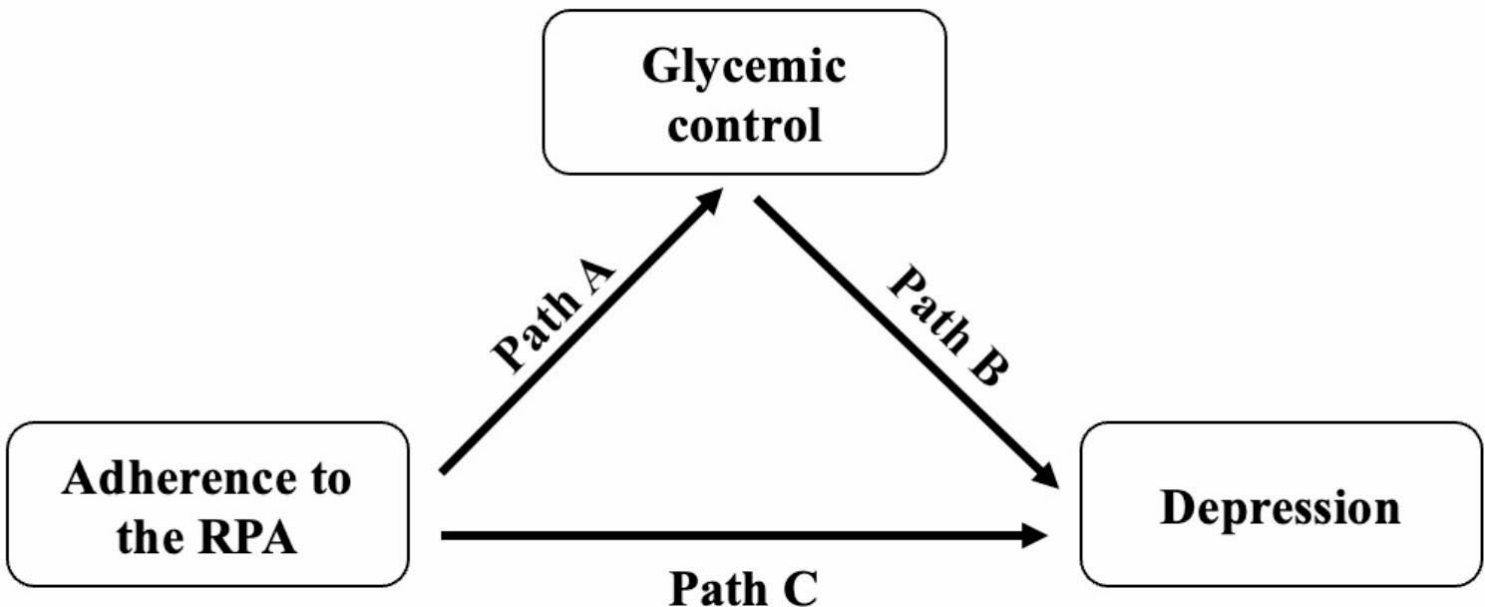
Path diagram of the mediation analysis model. RPA: recommended physical activity. Path a represented the relationship between adherence to RPA and glycemic control (mediator). Path b represented the relationship between glycemic control (mediator) and depression. Path c represented the direct relationship of adherence to RPA with depression. Path a together with path b indicated the indirect effect, and path c indicated the direct effect

To investigate whether glycemic control mediated the relationship between adherence to RPA and depression, three pathways (a, b, and c) were used to evaluate the mediation (Fig. 2). Path a was used to evaluate the relationship between adherence to RPA and glycemic control (mediator). Path b was constructed to assess the relationship between glycemic control (mediator) and depression. Path c was used to assess the direct relationship of adherence to RPA with depression. Total effect evaluated the relationship between adherence to RPA and depression. Path a together with path b indicated the indirect effect, and path c indicated the direct effect. In the mediation analysis, the indirect effect of physical activity on depression that goes through glycemic control was − 9.0 × 10^− 5^. The direct effect of physical activity on depression was − 2.9 × 10^− 2^ and the total effect was − 3.0 × 10^− 2^. Therefore, the proportion of the association of physical activity with depression that goes through glycemic control was 0.3% (indirect effect/total effect) and there was no statistical significance (P = 0.48). In the subgroup analyses by gender and age, the results were not statistically significant as well.

In addition, to test whether the association of adherence to RPA with depression differed among populations with different statuses of glycemic control, we performed a moderation analysis by adding a two-way interaction term between adherence to RPA and status of glycemic control into the regressions. In the moderation analysis, the p-value for the interaction term was 0.304.

## Discussion

Depression is one of the leading causes of mental health-related disease burden and a major cause of disability around the world. Prevention of depression is important to avoid unpleasant outcomes and effective interventions, including modification of established risk factors, are thus needed [32]. Both physically inactive and dysglycemia are established risk factors for depression [11–14]. However, their joint association with depression is not clear. Our study found that being physically active and achieving satisfactory glycemic control may both be important for the prevention of depression. Even if optimal glycemic control is not achieved, adherence to the RPA was associated with lower odds of depression, independent of metabolic risk factors such as comorbidities and insulin resistance. Alternatively, if individuals were not able to have adequate physical activity, then glycemic control may be beneficial in lowering the odds of depression. This result was further validated by the moderation analysis that the association of adherence to RPA with depression did not differ among populations with different glycemic control statuses.

In a recent dose-response meta-analysis of 15 prospective cohort studies, Pearce et al. found that individuals who adhere to the recommended volume of physical activity would have a 25% lower risk of depression, while there were diminishing additional benefits at higher volumes of physical activity. It was estimated that 11.5% of depression cases could have been averted if inactive adults had achieved the recommended volume [12]. However, this estimation was based on the general population without considering glycemic control, which was also an important risk factor for depression in patients with or without diabetes [13, 33]. It is important to examine whether physically active individuals would have reduced odds of depression, even if their HbA1c level remains elevated. Our studies extend the current knowledge and demonstrate that for all levels of HbA1c, those who adhere to the recommended RPA would be less likely to have depression compared to the inactive participants who had the same level of HbA1c. Moreover, it is interesting to know that the beneficial effects of physical activity remained significant after adjusting for the traditional metabolic risk factors such as BMI, BP, lipid profile, and insulin resistance. These results together highlight the benefits of physical activity on depression even for patients with poor glycemic control.

The potential physiological mechanisms for the relationship of physical activity with depression are not fully clarified [34]. We performed mediation analyses to examine whether the association of physical activity with depression goes through glycemic control. Mediation analysis has been increasingly used in clinical studies to evaluate the underlying mechanisms of different diseases [35]. This epidemiological approach disintegrates the association of a risk factor with the outcome (e.g., physical activity with risk of depression) into a direct association (not through glycemic control) and an indirect association (through glycemic control). It helps us to find out the potential effective intervention strategies to improve the outcome. For example, if there is a substantial indirect association between physical activity and depression (through glycemic control), interventions for better glycemic control other than physical activity would be helpful to decrease the odds of depression. However, the proportion of the association of physical activity with depression that goes through glycemic control was 0.3% and the result was insignificant, suggesting that physical activity did not associated with depression through glycemic control. It further proved that physical activity and glycemic control were related to depression independently and jointly.

We performed subgroup analyses to examine whether the combined association of physical activity and glycemic control with depression differed by gender and age as both of them were important risk factors for depression [30, 31]. Our results showed that the combined associations were different between men and women as well as between young and old adults. It seems like women and old adults would benefit more from physical activity and glycemic control. These results are not unprecedented. For example, Zhang et al. found regular physical activity reduced symptoms of depression among both genders, but notably among women. The reason for this difference might be that there are gender differences in some sociodemographic factors which play differentiated roles in affecting depressive symptoms [36]. Another study also found that among individuals with diabetes, older adults might gain more health benefits in reducing depression risk by being physically active [37]. The underlying mechanisms for the gender and age differences need to be explored further.

The strength of the current study lies in the relatively large sample size of participants and the reliability of the NHANES data, which have been validated by numerous published literature. However, the limitations of this study should be noted. First, due to the lack of temporality in cross-sectional studies, we can only reveal the association rather than an effect. We can only prove there was a combined association of adherence to RPA and glycemic control with depression. Second, the evaluation of depression was based only on one self-reporting scale. Using a summed score on the PHQ-9 to define depression might not be appropriate enough and a professional clinical diagnosis without recall bias would be needed for a more accurate description. Third, in the mediation analyses, we assumed that there were no other unmeasured confounders and no confounding between physical activity and depression. Thus, we are not able to rule out the possible risk of bias due to unmeasured confounders [38].

In conclusion, using the nationally representative database from NHANES, this study demonstrated that there was a joint association of adherence to RPA and glycemic control with depression, especially in women and older adults. We did not find out evidence of glycemic control mediation on the pathway from physical activity to depression. Further prospective studies are needed to verify our results.

## Data Availability

The data are from NHANES, which is openly available at https://www.cdc.gov/nchs/nhanes/index.htm.

## List of abbreviations

BMI: body mass index
BP: blood pressure
CI: confidence interval
CVD: cardiovascular disease
HOMA-IR: insulin resistance
OR: odds ratio
PHQ-9: 9-item Patient Health Questionnaire
RPA: recommended physical activity
NHANES: National Health and Nutrition Examination Survey
